# Orbital fat swelling: A biomechanical theory and supporting model for spaceflight-associated neuro-ocular syndrome (SANS)

**DOI:** 10.3389/fbioe.2023.1095948

**Published:** 2023-02-09

**Authors:** Matthew A. Reilly, Steven E. Katz, Cynthia J. Roberts

**Affiliations:** ^1^ Department of Biomedical Engineering, The Ohio State University, Columbus, OH, United States; ^2^ Department of Ophthalmology and Visual Sciences, The Ohio State University, Columbus, OH, United States; ^3^ Ohio Neuro-Ophthalmology, Orbital Disease & Oculoplastics, Columbus, OH, United States

**Keywords:** microgravity, biomechanics, spaceflight-associated neuro-ocular syndrome (SANS), orbital fat, cephalad fluid shift

## Abstract

Spaceflight-Associated Neuro-ocular Syndrome (SANS) is a descriptor of several ocular and visual signs and symptoms which commonly afflicts those exposed to microgravity. We propose a new theory for the driving force leading to the development of Spaceflight-Associated Neuro-ocular Syndrome which is described *via* a finite element model of the eye and orbit. Our simulations suggest that the anteriorly directed force produced by orbital fat swelling is a unifying explanatory mechanism for Spaceflight-Associated Neuro-ocular Syndrome, as well as producing a larger effect than that generated by elevation in intracranial pressure. Hallmarks of this new theory include broad flattening of the posterior globe, loss of tension in the peripapillary choroid, decreased axial length, consistent with findings in astronauts. A geometric sensitivity study suggests several anatomical dimensions may be protective against Spaceflight-Associated Neuro-ocular Syndrome.

## Introduction

Spaceflight-Associated Neuro-ocular Syndrome (SANS) is a common condition affecting individuals exposed to microgravity with severity partially dependent on length of time in microgravity. While its features vary, the most common ocular findings include optic disc edema, cotton wool spots, horizontal choroidal folds, globe flattening, shortening of axial length, and hyperopic shift (Brunstetter, 2018). To date, these symptoms and signs have been considered independently since the same set of findings were not consistently evaluated with each mission. For example, up to 29% of astronauts were reported to experience globe flattening (Stenger et al., 2017), but this requires an imaging study to evaluate, which was not consistently performed at pre and post-flight time points. Initially, elevated intracranial pressure (ICP) was thought to play a major role in optic nerve head edema (Stenger et al., 2017), and the syndrome was called Visual Impairment due to Intracranial Pressure (VIIP). However, it has since been recognized that ICP elevation alone is insufficient and other factors would likely be involved to generate the induced optic disc edema and retinal changes that were observed (Brunstetter, 2018). It has also been suggested that a chronic low increase in ICP occurs, accompanied by a lack of the normal terrestrial loading and unloading cycle associated with a change in position from supine to standing, which is absent in a microgravity environment (Stenger et al., 2017). Therefore, in recognition that other mechanisms may play an important role, the syndrome was renamed SANS.

**FIGURE 1:**
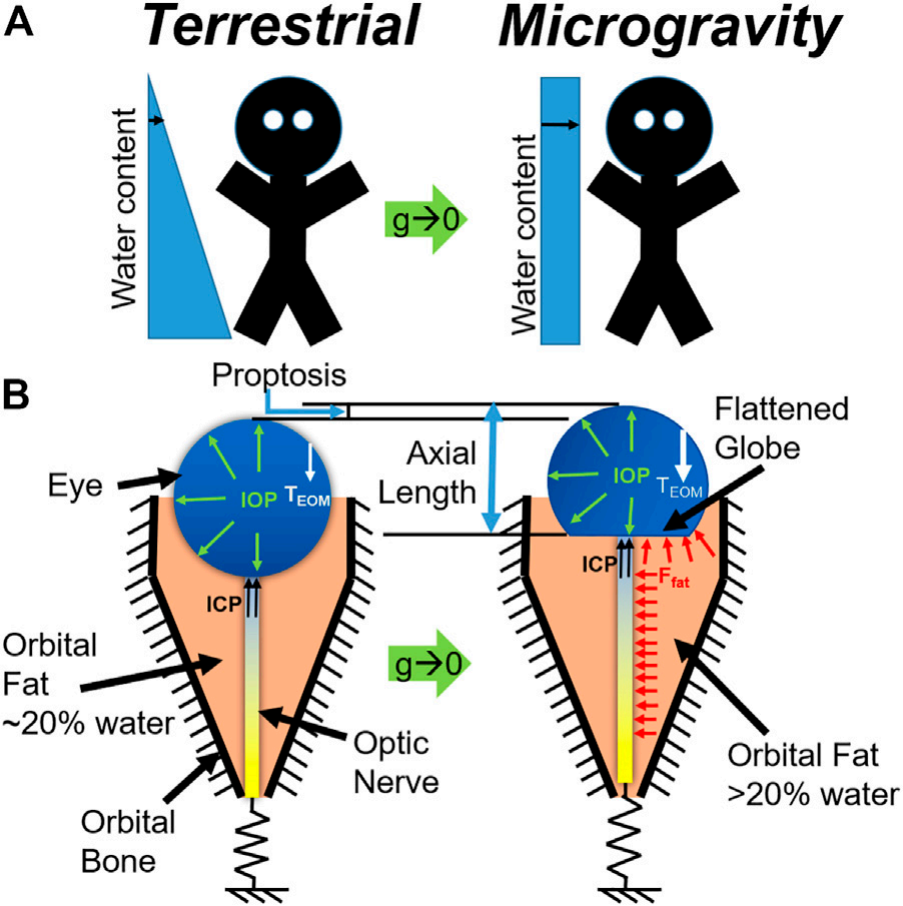
**(A)** Schematic representation of the microgravity-induced fluid shift and **(B)** corresponding changes in the eye and orbit (below). Model C is represented at left, while Model D is represented at right.

We present a mechanistic biomechanical theory, shown schematically in Figure 1, which unifies the ocular findings associated with SANS. It is proposed that during exposure to microgravity, the terrestrial hydrostatic pressure profile is absent, leading to a cephalad fluid shift. This increase in liquid volume would necessarily increase the volume of orbital contents and, therefore, apply an additional load on the globe (Louf et al., 2019). This additional load does not depend on hydrostatic potential but on water content. Another way to conceive of this is to consider the mechanical energy balance where the work done on the eye and orbit due to expanding orbital content volume is the integral of pressure over change in volume. Since the orbital bone is rigid relative to the eye, this work must be done on the eye itself. Thus, even if there is no change in orbital pressure, the amount of work done can be sufficient to deform the eye. This energy method is used in finite element analysis (Szabo and Babuska, 1991) to compute the equilibrium state of the orbital contents and eye and led us to our conclusion. We also note that there are several key differences between the terrestrial (supine) case and microgravity. In particular, the hydrostatic pressure gradient is never re-established during microgravity, implying that there may be some chronic component necessary for the equilibrium water distribution to shift so far in the superior direction.

**FIGURE 2:**
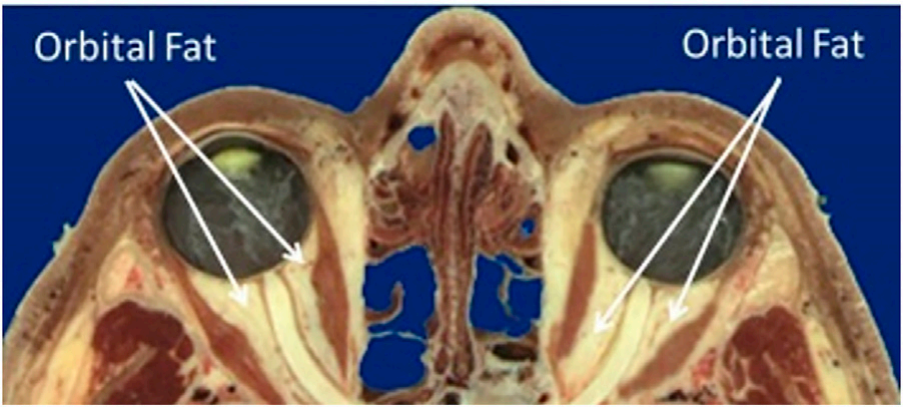
Sagittal section of the orbit and contents (Ackerman, 1998).

Figure 2 shows the anatomy of the bony orbit (Ackerman, 1998) which is shaped like a cone, gradually narrowing toward the apex. The retrobulbar space is filled with soft tissues including the optic nerve, ocular blood vessels, extraocular muscles, and adipose tissue or fat. The pressure exerted on the orbital structures is increased, including the bones defining the boundary of the orbit, the blood vessels entering the optic canal and tracking along the optic nerve, and the posterior surface of the globe involving the outer sclera, inner choroid, and retina. Therefore, the purpose of the current study is to computationally simulate the impact of increased orbital pressure on the ocular structures with finite element modeling and compare the predicted results to the ocular signs that have been reported with extended exposure to microgravity.

## Methods

### Theoretical basis

An axial force balance on the eye was first undertaken to define the system and estimate the relative contributions of each potential source to the axial motion of the eye. Specifically, these included contributions from intracranial pressure (*F*_*ICP*_), fat swelling (*F*_*fat*_), extraocular muscle tension (*F*_*EOM*_), and optic nerve stretching (*F*_*ON*_), giving the net force balance

$${↑}_{anterior}^{+}\sum F_{z}=F_{ICP}+F_{fat}-F_{EOM}-F_{ON}=0.$$ (1)

The homeostatic, terrestrial contributions from each source were estimated from literature sources and clinical experience as follows:

• *F*_*EOM*_ ∼ 1–4 mN in the posterior direction [passive contribution only; (Guo et al., 2016)]
• *F*_*ON*_ ∼ 0 for small displacements due to slack in the nerve; and
• *F*_*ICP*_ ∼ 0–1.75 mN [0–20 cmH_2_O, representing intracranial hypotension to borderline hypertension (Swyden et al., 2021)]

Based on this analysis, we are left to conclude that Eq. 1 can be reduced to

$$F_{EOM}\approx F_{fat}+F_{ICP},$$ (2)

Indicating that EOM tension must increase by some amount unknown *a priori* and depend on the balance of volumetric swelling forces and must be estimated computationally. The model also has a boundary condition representing *F*_*ON*_ for completeness since these effects may come into play at larger deformations or during eye movements.

### Model construction

**FIGURE 3:**
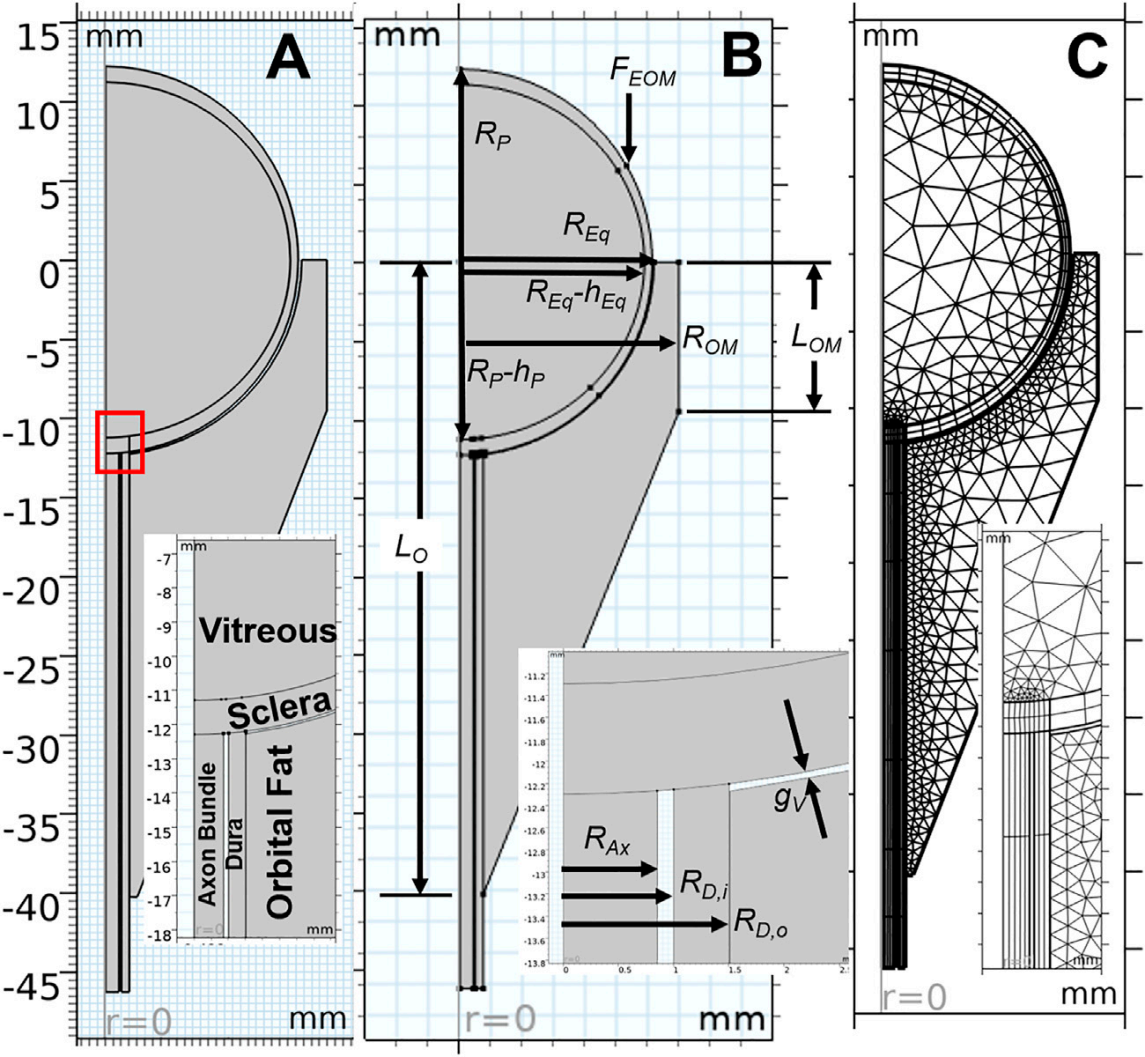
Key aspects of the computational model are shown, with inset images detailing the optic nerve head region. An axisymmetric representation of ocular and orbital anatomy is given in **(A)**, while **(B)** shows parameterization of the geometry. Note that all parameter values for the base model are given in Table 1. The finite element meshes used to solve the Baseline Model are shown in **(C)**.

Based on the force balance, an axisymmetric geometric model (Figure 3) was constructed in COMSOL Multiphysics v5.6 (COMSOL, Inc., Burlington, MA) using nominal dimensions for the eye and orbit, based on the histological sections in Figure 2 (Ackerman, 1998). A baseline model intended to represent a normal human eye and orbit, in terms of anatomy and material properties was created and used as a basis for modeling predictions (Figure 3A). This model, referred to as the “Baseline Model,” was used as a starting point for all sensitivity analyses performed. This geometry was parametrized as shown in Figure 3B, while Table 1 gives the parameter values for the Baseline Model. An ICP of 10 mmHg (13.6 cmH_2_O) was chosen for the Baseline Model as it corresponds to the middle of normal ICP [6.5–19.5 cmH_2_O (Swyden et al., 2021)].

**TABLE 1:** Geometric parameters used for the baseline model.

Symbol	Description	Value	Source
*R*_*Eq*_	Equatorial radius	12.36 mm	Figure 2
*R*_*P*_	Polar radius	12.36 mm	Figure 2
*h*_*Eq*_	Equatorial thickness	0.5 mm	
*h*_*P*_	Polar thickness	1.0 mm	
*R*_*OM*_	Orbit margin radius	13.95 mm	Figure 2
*L*_*OM*_	Orbit margin length	9.51 mm	Figure 2
*L*_*O*_	Total length of orbit	40.26 mm	Figure 2
*R*_*A*_	Radius of axon bundle	0.85 mm	Lagrèze et al. (2009)
*R*_*D,i*_	Inner radius of dura	1.0 mm	Figure 2
*R*_*D,o*_	Outer radius of dura	1.515 mm	Lagrèze et al. (2009), Figure 2
*g*_*v*_	Gap to allow for vitreous swelling	133–217 µm	From simulation

The living eye is loaded with intraocular pressure (*IOP*), intracranial pressure (*ICP*), tension from the extraocular muscles (*F*_*EOM*_), and the contact force generated by the swelling fat (*F*_*fat*_). All tissues were modeled using an incompressible, isotropic, neo-Hookean hyperelastic material model. Assumed values for elastic moduli are given the table below.

**FIGURE 4:**
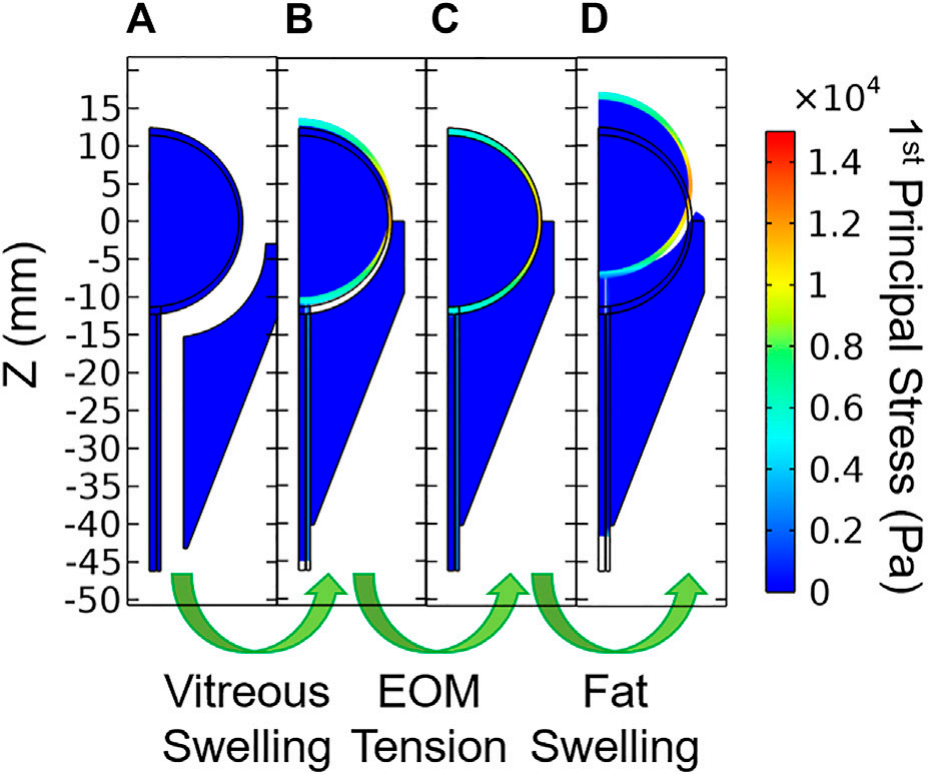
Stages of model loading. **(A)** The zero-stress state is determined iteratively, then vitreous swelling is used to produce **(B)** the residually-stressed state of the eye. Extraocular muscle tension is then incremented which gives **(C)** the *in vivo* terrestrial geometry. Finally, orbital fat swelling is simulated to produce **(D)** the predicted ocular dimensions in microgravity.

Each model was solved using a sequence of three steps (Figure 4). Model A modeled the zero-stress state of the eye and optic nerve in the presence of vitreous swelling and ICP while the orbit, fat, and all other external forces were absent. Model A was found using a coordinate search algorithm by varying the geometry of the zero-stress eye (i.e., the polar and equatorial elliptical radii) and the vitreous hygroscopic strain such that the resulting eye corresponded to the target geometry and IOP. Loading of Model A with vitreous swelling gives the residual stress state of the eye, Model B. Model B was then taken as the initial condition of the eye and optic nerve when simulating fat swelling as follows. Model B was set within the orbit and orbital fat, then the extraocular muscle tension was gradually added to find Model C—the terrestrial (homeostatic) state of the eye and orbit. Finally, orbital fat swelling and ICP were incremented to study the response of the eye and optic nerve, giving Model D as a final output. Results described below are for Model D relative to Model C rather than the intermediate models; it therefore represents the changes which might occur due to the hypothesized swelling of the orbital contents arising from the microgravity-induced fluid shift.

Fat swelling was modeled using a hygroscopic swelling model in which the hygroscopic volume ratio $J_{H}$ was varied as a modeling parameter. This was achieved in COMSOL by using the hygroscopic swelling model, where the hygroscopic strain $\varepsilon_{H}=\beta \left(C_{W}-C_{W,0}\right),$ with *β* as the coefficient of hygroscopic swelling of the fat-water mixture (equivalent to the inverse of density, or about 0.001 m^3^/kg for water), *C*_*W,0*_ as the baseline (terrestrial) water content, and *C*_*W*_ as the current water content. Thus, $J_{H}={\left(1+\varepsilon_{H}\right)}^{3}$ . Values of *J*_*H*_ were studied to indicate how much fat swelling would be required to induce the ocular findings associated with SANS.

The IOP was also generated using a hygroscopic swelling model to mimic the physiological process which maintains IOP (i.e., the eye has more fluid in it than it does in its zero-stress state). In this case, the dimensions of the zero-stress geometry were estimated, swelling in the vitreous simulated, and the difference between the model predictions and target ellipse radii and target IOP were calculated using convex objective functions. COMSOL’s Optimization Module was used to determine the zero-stress geometry, the amount of “swelling” required to achieve IOP. Once this optimization was complete, the orbit and fat were constructed and assumed to be in contact with the eye and ON prior to simulating fat swelling.

### Boundary conditions

This residually loaded state was then inserted into the model containing the fat, *F*_*EOM*_, and ICP to find the terrestrial baseline geometry and stresses. The lateral and posterior boundaries of the orbital fat were held fixed to simulate attachment to the orbital bone. Intracranial pressure was applied to the exterior surface of the sclera over a segment representing the sub-arachnoid space with a radial distance of 270 µm [estimated from Figure 1 of (Lagrèze et al., 2009)].

Contact boundaries were established between the orbital fat and the sclera. This allowed sliding to occur at the sclera-fat interface, as would be expected *in vivo* as the eye must easily rotate during routine visual tasks.

Boundary conditions were applied as indicated in the free body diagrams shown in Figure 1B. The nerve is constrained using a spring boundary condition: the tension in the nerve increases with forward translation of the eye. Thus, tension could arise in the nerve if (A) significant proptosis occurred and/or (B) normal eye movements resulted in removal of slack. Demer (Demer, 2016) measured ON length in relation to orbit length, finding an increasing trend [Figure 11 of (Demer, 2016)]. To infer slack from these measurements, one would also need to know the axial length of the eye, allowing the slack to be approximated as

$$ON\;slack=ON\;length-orbit\;length-\frac{1}{2}\left(axial\;length\right).$$

Taking the values for orbit length and axial length from Table 1, along with the regression equation from the referenced figure, gives a value for ON slack of 8.4 mm. We therefore chose a threshold slack of 6 mm as a conservative estimate of ON slack. This threshold was never exceeded during any simulations, so the optic nerve remained slack at all times.

The extraocular muscle tension was applied as a point source to the peripheral anterior globe. This tension was modeled as an analytical function representing the force required to achieve passive stretching of a single rectus muscle multiplied by the number of rectus muscles (i.e., four) [Eq. 10 of (Guo et al., 2016)]:

$$F_{EOM}=4M\left(-0.29w+4.79e^{\frac{w}{4.57}}-3.01\right),$$ (3)

where *w* is the anterior displacement of the EOM insertion point in mm to give *F*_*EOM*_ in mN and *M* is a multiplier used to increment the loading as described below. Generally, *M* was incremented from 0–1 to simulate the passive muscle tension. Since the active forces in the muscle are unknown, an additional study was undertaken in which *M* was incremented from 0–2.25 to simulate additional EOM tension arising from active contributions. This simplifying approach was adopted since neither the active nor passive contributions are known for SANS, and an *M* value of 2.25 resulted in a net doubling of *F*_*EOM*_.

### Simulations

The balance of forces was computed numerically using finite element analysis in COMSOL Multiphysics (Figure 3) to investigate the effect of an increase in retrobulbar fat water content on the eye. All simulations included geometric non-linearity. Following the aforementioned procedure for determining the terrestrial state of the eye, the ICP and *J*_*H*_ were systematically varied to simulate the effects of a microgravity-induced fluid shift on the eye and orbital tissues.

Changes to axial length and posterior radius of curvature (ROC) were calculated as a proxy for globe flattening. ROC was estimated by fitting a circle to the deformed scleral surface within a 4 mm radius of the axis of symmetry using the Pratt method (Pratt, 1987). Change in choroidal arc length was used to imply the formation of choroidal folds: shortening indicates a decrease in tension and the possibility of wrinkling (Ligarò and Barsotti, 2008; Barsotti and Ligarò, 2014). The peripapillary arc length is the arc length along the inner boundary of the sclera spanning the posterior-most quarter of the eye’s circumference in the zero-stress state. While this definition is somewhat arbitrary, we found that the results were robust when other definitions were considered (e.g., 1/8 or 1/2 rather than 1/4).

An additional series of simulations was conducted to determine whether and to what extent measurable anatomical variations may influence the risk of SANS based on a change in the water content of the orbital fat with exposure to microgravity. The varied parameters were polar radius *R*_*p*_ (where axial length = *2R*_*p*_), equatorial radius *R*_*eq*_, orbit depth *L*_*0*_, radius of the orbit margin *R*_*OM*_, and depth of orbit margin *L*_*OM*_ as shown in Figure 3B.

## Results

### Model verification

Iterative mesh refinement indicated that the solution was largely independent of mesh density using quartic Lagrangian shape functions, even with a coarse mesh. The mesh density used for the reported simulations (Figure 3C) was therefore selected on the basis that it offered improved stability for the non-linear solver for the variety of studies conducted.

### Baseline model predictions

**FIGURE 5:**
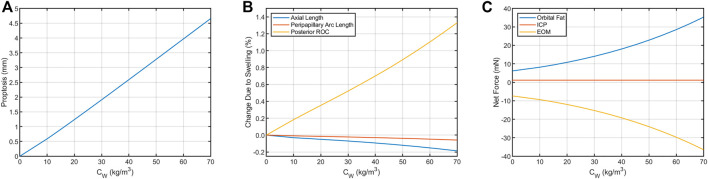
Resultant axial loads predicted for each term in Eq. 2 for the Baseline Model. In the terrestrial state (hygroscopic strain = 0), tension in the extraocular muscles (EOM) keeps the eye seated in the orbit by pulling in the posterior direction. This tension is balanced by the net axial load arising from intracranial pressure (ICP, assumed to be 13.6 cmH_2_O) and compression of the orbital fat. This results in **(A)** proptosis and **(B)** decreases in axial length and peripapillary arc length along with increased posterior ROC. **(C)** These biometric changes are the result of changes in the balance of forces between the eye, fat, and extraocular muscles. The contribution from ICP increases from 1.17 to 1.75 mN if ICP increases from 13.6 cmH_2_O to 20 cmH_2_O, representing a possible increase from normal to borderline intracranial hypertension (Swyden et al., 2021).

The predicted deformations corresponding to 0%–7% increase in retrobulbar content volume are given in Table 2 and shown in Figure 5. Decreasing axial length and increasing posterior ROC correspond to physical signs of globe flattening. Decreased peripapillary arc length indicates compression of, or reduced tension in, the choroid which could result in choroidal folds. In addition, the model predicted low levels of proptosis. Although subtle proptosis has not been reported with SANS, it could be easily missed on clinical exam.

**TABLE 2:** Changes in ocular anatomy due to orbital fat swelling.

Spaceflight-associated water in orbital fat (kg/m^3^)	Orbital fat volume ratio (dimensionless)	Proptosis (mm)	Change in axial length (µm)	Change in peripapillary arc length (µm)	Change in posterior ROC (mm)
0.000	1.000	0.00	0.00	0.00	0.00
0.010	1.030	0.59	−7.29	−0.78	0.02
0.015	1.046	0.91	−9.56	−1.11	0.03
0.020	1.061	1.24	−11.81	−1.42	0.04
0.025	1.077	1.57	−14.16	−1.74	0.06
0.030	1.093	1.91	−16.66	−2.06	0.07
0.035	1.109	2.25	−19.33	−2.40	0.08
0.040	1.125	2.59	−22.20	−2.76	0.09
0.045	1.141	2.93	−25.26	−3.13	0.10
0.050	1.158	3.28	−28.58	−3.54	0.11
0.055	1.174	3.62	−32.12	−3.96	0.13
0.060	1.191	3.97	−35.94	−4.41	0.14
0.065	1.208	4.31	−40.03	−4.89	0.15
0.070	1.225	4.66	−44.41	−5.39	0.17

### Sensitivity analysis

**FIGURE 6:**
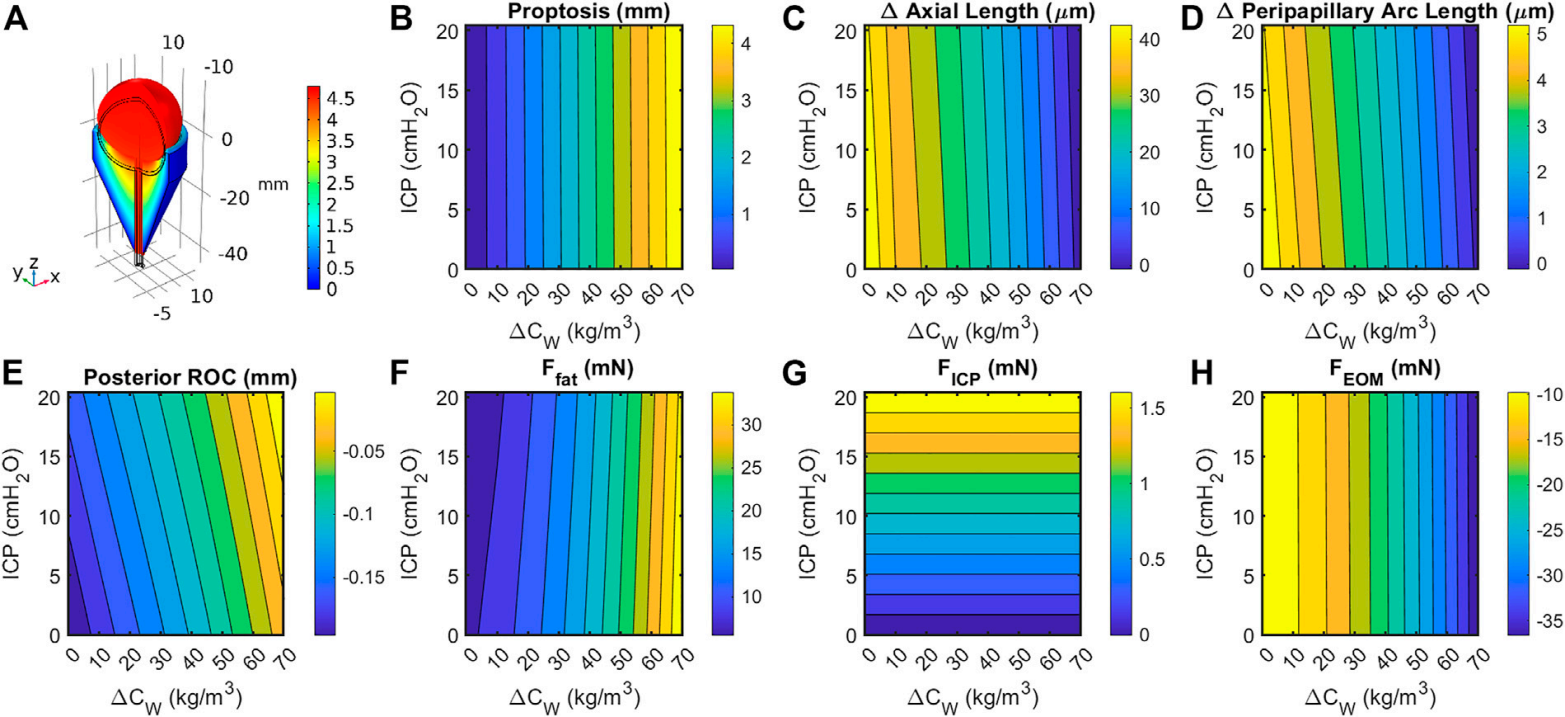
**(A)** Cutaway section showing eye, optic orbit, and optic nerve following a 15% increase in retrobulbar fat volume. Black lines indicate geometry prior to swelling. Coloration indicates total change in volume due to elastic and hygroscopic effects. Fat swelling, but not ICP, had a large effect on change in **(B)** axial length; **(C)** posterior radius of curvature within 4 mm of axis; **(D)** peripapillary arc length decreases, suggesting decreased tension and possibly choroidal folding; and **(E)** proptosis of the anterior pole. Contributions to the axial force balance (Eq. 2) from **(F)** fat swelling, **(G)** intracranial pressure, and **(H)** extraocular muscle tension are largely one-dimensional, though the fat force is slightly dependent on both intracranial pressure and fat volume ratio.

Sensitivity studies were performed to evaluate the model’s sensitivity to modeling assumptions. First, the boundary conditions were investigated. Since a single representative ICP value selected for the Baseline Model was selected from numerous literature values, a thorough sensitivity analysis was performed to compare the relative contributions of ICP and fat swelling to ocular deformation (Figure 6). Axial length, posterior ROC, peripapillary choroidal arc length, and proptosis depend very weakly on ICP but strongly on orbital fat swelling. Applying IOP using a hygroscopic swelling model in the vitreous gave results which were nearly identical (i.e., within 1%) to those applying a pressure boundary condition to simulate IOP. Initial simulations modeled the boundary between the eye and ON with the orbital fat as a tied boundary, as opposed to the more realistic frictionless contact boundary. This caused much larger ocular deformations but gave qualitatively similar results in all cases. Approximately doubling *F*_*EOM*_ to simulate active tension in the EOM decreased proptosis by 7% while resulting in a 63% larger drop in axial, 33% more decline in peripapillary arc length, and increased radius of curvature by 8%. This was achieved by setting *M = 2.25* in Eq. 1, thereby increasing the maximum EOM force from 25.3 to 53.8 mN for the same extent of swelling.

**FIGURE 7:**
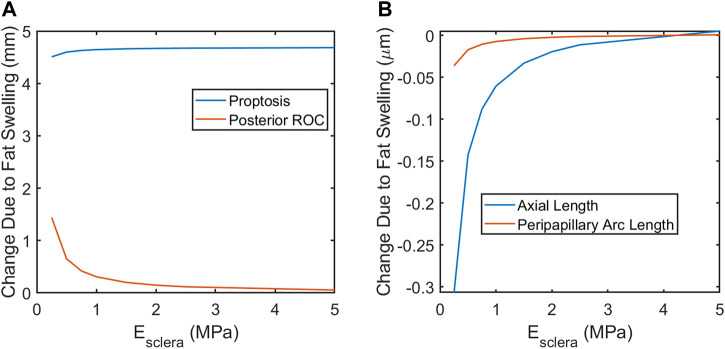
A softer sclera may result in increased magnitude of SANS-associated changes. **(A)** proptosis is relatively independent of the scleral elastic modulus. Globe flattening, as indicated by increased posterior ROC **(A)** and decreased axial length **(B)**. Severity of choroidal folding, as indicated by decreased peripapillary arc length, would be higher in softer eyes.

Next, the elastic moduli *E* of the sclera, vitreous, and fat were varied over a broad range. Varying *E*_*fat*_ from 0.7–1.5 kPa and *E*_*vitreous*_ from 6.5–25 Pa had no effect on any modeling predictions over the entire range of *E*_*sclera*_ considered (0.25–5 MPa) as expected, since their incompressibility dominates their mechanical response. The fat and vitreous have a very low shear modulus all materials were modeled as incompressible and linearly elastic with the properties given in Table 3. Relative to bulk modulus, implying that they will effectively behave as incompressible fluids under the conditions studied: they change shape very easily but do not appreciably change volume due to loading. However, the response of the eye depended strongly on *E*_*sclera*_ over this range, with all SANS signs increased in magnitude in less stiff eyes (Figure 7).

**TABLE 3:** Tissue properties used for baseline model.

Tissue	Elastic modulus	Source
Ocular coats	1.5 MPa	Nguyen et al. (2018)
Vitreous humor	6.5 Pa	Tram and Swindle-Reilly (2018)
Orbital fat	700 Pa	Yoo et al. (2011)
Orbital bone	Infinite (rigid)	—

**FIGURE 8:**
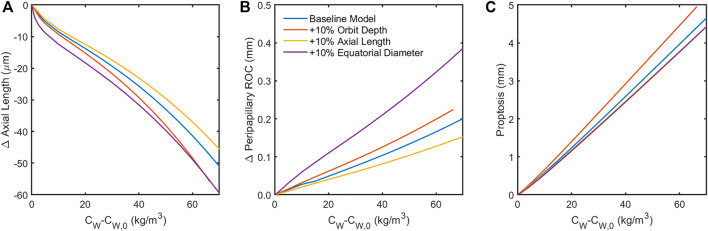
Sensitivity of the model to 10% increase in anatomical dimensions. Increasing the orbit depth had the largest effect of all orbital dimensions, while increasing the equatorial diameter of the globe had the largest effect of all ocular dimensions. Both of these parameters had the effect of increasing the force of fat swelling on the globe’s posterior by placing more fat behind the eye (orbit depth) or confining the space around the eye available for anterior displacement of the swelling fat (equatorial diameter). Increased axial length appears to offer some protection from globe flattening **(A,B)**. Note that the results in **(C)** for changes in axial length and equatorial diameter are indistinguishable, suggesting that proptosis is diminished when the eye is larger owing to displacement of orbital fat volume.

Finally, sensitivity to orbital anatomy and ocular anatomical dimensions were examined (Figure 8). The model predicted orbit depth (*L*_*O*_) to be the most important feature of orbit anatomy, with a 10% increase resulting in ∼25% changes in the predicted responses. Increasing the eye’s equatorial diameter by 10% had a similar effect on globe flattening (i.e., change in axial length and posterior radius of curvature). Orbital depth varies considerably between individuals. Fat swelling-induced changes could therefore depend significantly on an individual’s orbital anatomy.

The terrestrial force balance (Eq. 2) for the Baseline Model indicates that $F_{EOM}\approx 7.36\;mN$ , $F_{ICP}\approx 1.17\;mN$ , and $F_{fat}\approx 5.6\;mN$ . Increasing ICP to 20 cmH_2_O–the lower limit of borderline intracranial hypertension (Swyden et al., 2021)—only increased $F_{ICP}\approx 1.75\;mN$ , while 7% swelling could increase $F_{fat}\approx 36.3\;mN$ . Thus, even a pathological increase in ICP would be unlikely to contribute to a biomechanical mechanism of SANS.

## Discussion

**FIGURE 9:**
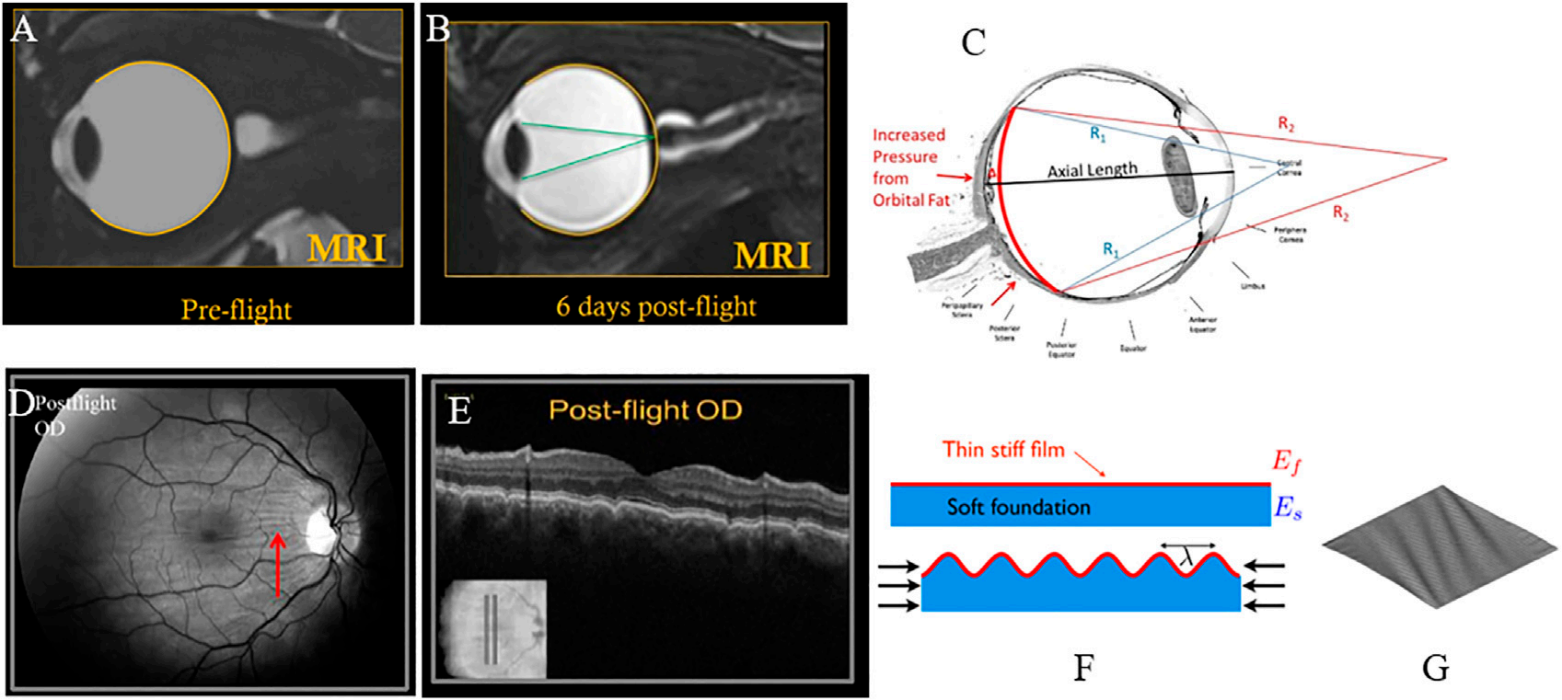
**(A)** Pre-flight MRI of astronaut; **(B)** Post-flight MRI with globe flattening and shortened axial length leading to hyperopic shift with focus of light behind the retina (Brunstetter, 2018). **(C)** Arclength of the sclera shortens with globe flattening, as illustrated by the red curve [adapted from (Gogola et al., 2018)]. **(D)** Choroidal folds in an astronaut measured post-flight; **(E)** corresponding cross-sectional OCT image showing undulations or waviness (Gogola et al., 2018). **(F)** Schematic illustration of wrinkling of a thin film with forces that shorten the foundation (Holmes, 2015). **(G)** Plot of a thin membrane subject to shear forces, generating wrinkles (Diaby et al., 2006). All figures **(A–E)** reused under CC BY-NC-ND license. **(F)** is reused with written permission of the author. **(G)** reused under CC BY license from HAL Open Science.

As orbital fat water content increased, the model predicted a small amount of forward motion of the eye (proptosis), a shortening of axial length which is associated with globe flattening, and negative choroidal strain that would be associated with induced choroidal folds. The change in axial length, globe flattening, and choroidal folds are consistent with ocular findings in astronauts that have been reported and are illustrated in Figure 6. Up to 29% of astronauts have experienced globe flattening [Figure 9; (Brunstetter, 2018)], as well as a reported hyperopic shift. A hyperopic shift result from shortening of axial length. Choroidal folds occur in 21% of astronauts during long duration space flight [Figures 9D, E; (Brunstetter, 2018)]. It can resolve or reduce post-flight, but persistence up to 12 years has been measured [Figure 9E; (Brunstetter, 2018)]. The model predicts that as the globe flattens under the increased pressure exerted by the swelling orbital fat, the arc length of the impacted section of the globe shortens [Figure 6; (Gogola et al., 2018)]. If the scleral arc length shortens enough, the choroidal membrane it supports wrinkles, as illustrated in the analogous case of thin films in Figures 9F, G (Diaby et al., 2006; Holmes, 2015).

Proptosis has not been previously reported in astronauts. However, it has likely never been adequately evaluated or quantified since it is not obvious due the relatively small magnitude in the presence of facial swelling. In thyroid-associated orbitopathy (TAO) (Kaichi et al., 2016) and Rosai-Dorfman disease with proptosis (Singh et al., 2018), an increase in volume of the orbital contents results in proptosis. Proptosis during thyroid eye disease has been found to exceed 2 mm in a significant proportion of patients (Dorkhan et al., 2006). Thus, it is reasonable to expect subtle proptosis may occur in a microgravity environment due to cephalad fluid shift. A benefit of this numerical model is that it suggests a testable hypothesis that would add supporting evidence if found in astronauts. In addition, optic disc edema has been reported in thyroid eye disease which resolves with decompression surgery (Kleinberg and Bilyk, 2016), similar to astronauts. Patients with active TED have experienced a hyperopic shift as their orbital pressure increases, also similar to astronaut experience and predicted by our model. In addition, flattening of the posterior globe is reported in TED due to increased volume of orbital contents (Chandrasekaran et al., 2006).

Elevated intracranial pressure has been considered as a possible risk factor for SANS (e.g., Feola et al., 2016; Raykin et al., 2017). The present model suggests that, while ICP elevation could play a role, its biomechanical effects directly contributing to SANS pathology is likely limited. This does not rule out ICP elevation as a potential mechanobiological contributor to SANS that the present model cannot predict.

One key result of this study is the identification of possible anatomical risk factors which might increase sensitivity of astronauts to SANS. In particular the depth of the orbit can have a very large effect on all SANS signs, with larger orbital depth leading to greater shortening in axial length, greater globe flattening leading to increased risk of choroidal folds, and greater proptosis. In contradistinction, longer axial length may be protective against specific aspects of SANS, including less globe flattening.

**FIGURE 10:**
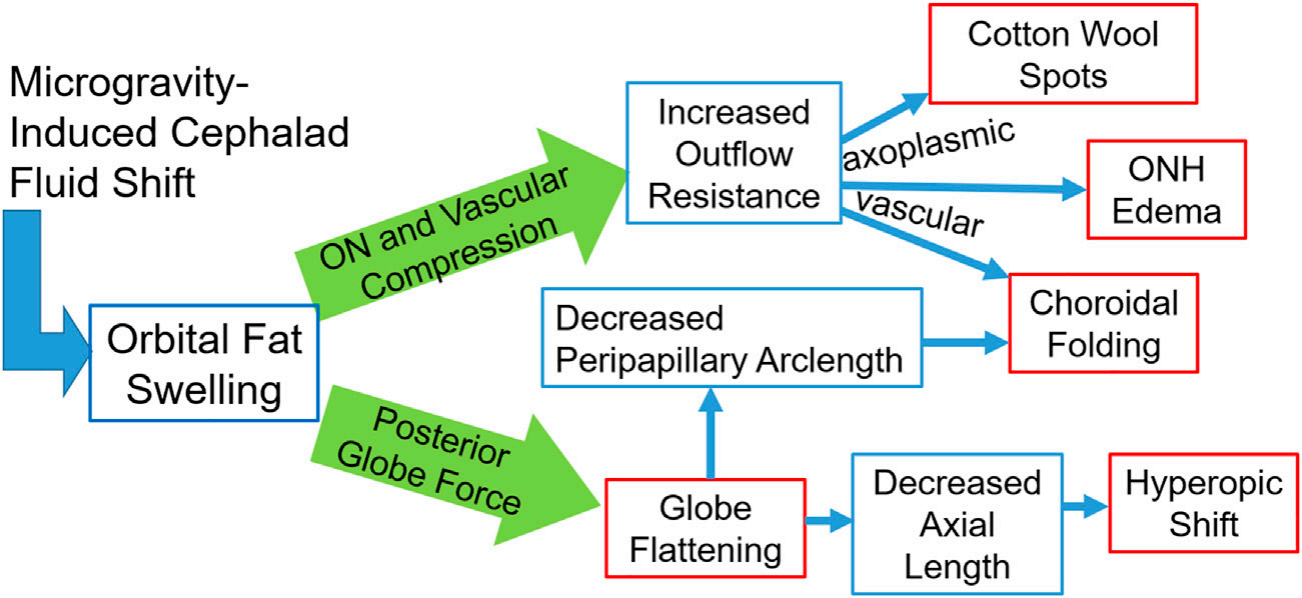
A schematic representation of the fat-swelling theory of SANS. Orbital fat swelling would necessarily lead to increased radial compression of the optic nerve and orbital vasculature, thereby increasing both axoplasmic and vascular resistance. These changes would present as cotton wool spots, ONH edema, and choroidal folding (in conjunction with globe flattening). Fat swelling will also cause a distributed loading applied to the globe’s posterior surface, resulting in decreased axial length and/or proptosis. Globe flattening would likely contribute to the formation of choroidal folding as well as a hyperopic shift. Red boxes indicate the signs and symptoms comprising SANS, while blue boxes indicate the hypothesized biomechanical linkages between the cephalad fluid shift and SANS.

Although not directly modeled, it is expected that with orbital congestion, the low-pressure venous outflow through the ophthalmic vein may be compromised. This would be expected to result in a mild chronic increase in venous pressure. A comprehensive schematic of the proposed effects of orbital congestion is given in Figure 10. The posterior globe force arm has been supported by the results of the presented biomechanical simulations. The optic nerve and vascular compression arm is hypothesized to account for the remaining ocular findings, such that orbital congestion is a unifying mechanism underlying most reported signs and symptoms of SANS.

One significant limitation of this model is its sensitivity to the relationship between anterior motion of the globe and resulting EOM tension. The EOM tension in the present form of the model is taken from the passive contribution to stretching a fully relaxed rectus muscle. In reality, some unknown active contribution to the EOM tension would be present under most circumstances. Our computational investigation shows that incorporation of this muscle activation decreases proptosis and enhances globe flattening. Without a more detailed knowledge of EOM activation, this model can still place constraints on the upper and lower limits by considering the two extreme cases where the muscle is purely passive (as in the Baseline Model) and the muscle is rigid (i.e., it prevents all anterior translation of the globe). The true case must then lie somewhere between.

Choroidal folding presumably results from a loss of tension in the direction perpendicular to wrinkling. This is a geometric phenomenon and would therefore be driven by globe flattening: as the principal radii of curvature change, tension in an inflated membrane (e.g., the choroid) can be lost (Burd et al., 2017). Once tension is removed, these folds may be permanent (Cassidy and Sanders, 1999).

Finally, the model suggests the importance of scleral stiffness and various anatomical markers in predicting susceptibility to SANS. In particular, lower scleral stiffness, shorter axial length, greater equatorial diameter, and greater orbital depth all increase susceptibility to globe flattening and the subsequent hyperopic shift and choroidal folds as orbital fat swells. Recent studies have indicated the possibility of extracting the biomechanical properties of the sclera *in vivo* from air-puff tonometry data (Nguyen et al., 2020). Therefore, scleral stiffness and axial length are readily measured with existing ophthalmic clinical devices, while equatorial diameter, orbital depth, and baseline posterior globe curvature could be quantified with an MRI.

The primary limitations of this model are due to its axisymmetry, crude anatomical representation, the use of simple material models, and lack of active contributions to EOM tension. For example, since this is an axisymmetric model, it cannot predict the directionality of choroidal wrinkles, but suggests the mechanical mechanism driving them: wrinkles in the horizontal direction suggest a loss of tension in the superior-inferior direction. Still, none of these factors is likely to alter the underlying mechanism of SANS presented in this study.

If this model is to be adapted to predict SANS susceptibility in astronauts, future studies should include expanding this model to three dimensions. Inclusion of subject-specific anatomical measurements and biomechanical properties, especially of the sclera, would allow screening for risk or prophylactic treatment. Pre-flight MRI data could supply the requisite anatomical information.

## Conclusion

This biomechanical theory for orbital fat swelling-induced SANS can explain all reported signs of SANS using a single mechanism. The other hallmark prediction of this theory–proptosis–has not been reported but this may be a result of its subtlety in concert with facial swelling due to the microgravity-induced headward fluid shift. Measurements of ocular position (i.e., proptosis) and shape, as well as orbital fat volume and water content before, during, and immediately after spaceflight would offer a powerful validation of this theory. In addition, an understanding of the mechanism would allow development of effective countermeasures. Greater understanding of individual characteristics (i.e., orbital depth, axial length, scleral stiffness) associated with susceptibility to SANS is paramount.

## Data Availability

The original contributions presented in the study are included in the article/Supplementary Material, further inquiries can be directed to the corresponding author.
